# Immediate dam-sourced colostrum provision reduces calf mortality - management practices and calf mortality in large dairy herds

**DOI:** 10.1186/s13028-024-00780-8

**Published:** 2024-12-05

**Authors:** Steffi Keller, Karsten Donat, Stefanie Söllner-Donat, Axel Wehrend, Anne Klassen

**Affiliations:** 1 Clinic for Reproduction Medicine and Neonatology of Animals, Faculty of Veterinary Medicine, Justus-Liebig-University Giessen, Frankfurter Str. 106, 35392 Giessen, Germany; 2 Thuringian Animal Disease Fund (institution by law, Animal Health Service, Thüringer Tierseuchenkasse AdöR, Victor-Goerttler-Straße 4, 07745 Jena, Germany

**Keywords:** Calf feeding hygiene, Calf losses, Calf management, Colostrum management, Dust

## Abstract

**Background:**

Farm-specific management practices greatly impact calf mortality rates. This cross-sectional study aimed to analyse the association between calf mortality and management practices in large dairy farms. A total of 93 dairy farms were voluntarily included in the study. All farms reared their own youngstock, and all but one kept more than 100 dairy cows. From March 2017 to March 2018, calf management practices were monitored during a farm visit, and farm managers were surveyed regarding calving procedures, neonate management, and environmental factors. Data were collated and analysed in conjunction with the 2017 calf mortality rate, as determined for each farm by using data from the German database of animal origin and movement (HI-Tier). All variables from the topics of colostrum supply, calf feeding, housing, health related information and calving preparation of the cows that resulted in *P* ≤ 0.1 in the analysis of variance were assumed to be associated with the calf mortality rate and were considered for a general linear mixed regression model.

**Results:**

According to the data from the HI-Tier database of the 93 study herds from 2017, 54,474 calves were born alive and 3,790 calves died within the first six months of life. The calf mortality rate was lower on farms where calves were immediately provided with dam-sourced colostrum. Farm managers perceiving dust as the primary factor precipitating respiratory disease on the farm was positively associated with calf mortality. Regularly replacing bucket teats correlated with lower calf mortality rates compared to replacing them only upon detection of abrasion.

**Conclusions:**

The study findings suggest that feeding calves with dam-sourced colostrum can potentially reduce overall calf mortality within the herd. This management practice holds comparable importance to ensuring successful passive transfer through timely and adequate colostrum feeding. Moreover, maintaining a low dust environment for the calves and consistently replacing bucket teats play significant roles in promoting better overall calf health.

## Background

Calves that mature into productive and longevous dairy cows form the foundation of sustainable dairy farming and breeding. To establish economically stable and efficient dairy farms, farmers must raise healthy calves exhibiting rapid development [1, 2]. A study in Belgium, France and the Netherlands calculated a median value of losses due to mortality of 508 € in 2018, calculated as the number of dead calves per farm in the first 3 weeks of life, multiplied by the average live calf sale price at that time for a standard weight [3]. Furthermore, besides the economic impact of calf mortality, the calf mortality rate is increasingly considered a metric for assessing animal welfare on farms [4]. Given the substantial rise in public awareness regarding animal welfare and protection in recent years, as shown by previous empirical studies [5–7], there has been a sharp decline in societal acceptance of intensive livestock farming, even if dairy cattle farming is viewed somewhat less critically by the general public than pig and poultry farming [8]. Previous studies have shown that social perceptions of agricultural animal husbandry are usually in conflict with current practice [6, 7]. The public is therefore calling for scientifically based guidance on the implementation of animal welfare in agricultural animal husbandry systems, in order to provide these animals with a basic standard of welfare [9].

The average calf mortality risk in countries with intensive milk production ranged from 3.3−5.3% during the last two decades, for instance with 3.5% in Canada [10], 5% in the United States of America [11] and 4.6% in Norway [12]. However, data collected over recent decades reveal substantial global and national variability in calf losses, spanning from 5−15% [13]. The calf mortality risk varied in Germany during the last two decades. During the 2001/2002 reporting period, Mecklenburg-Western Pomerania recorded a calf loss rate of 9.4% [14], while Bavaria observed a rate ranging from 12 to 14% in 2004 [15]. In 2012, calf losses in northeast Germany averaged at 5% [1]. Contrastingly, Lower Saxony documented a calf mortality rate of 8.1% in 2016 [16]. Thuringia recorded an average calf loss rate of 7.7% in 2006/2007 [17]. Although Thuringia’s average calf mortality rate remained relatively stable in 2016 (7.4%), only 27% of farms reported losses of less than 3%, while 19% of farms recorded losses exceeding 10% [18]. These figures significantly surpass the recommended maximum target value of 5%, as indicated in the literature [4, 13].

Approximately half of all calf fatalities are attributed to diarrhoea, with an additional quarter linked to respiratory disease [13]. The analysis of factors impacting calf mortality has highlighted the substantial influence of management practices on the scale of these losses. Particularly during the rearing phase, pivotal factors include the initial provision of colostrum, calving hygiene, housing type (individual/group housing, warm/cold climate housing), watering and feeding protocols, and hygiene practices [1, 2, 13, 15]. Colostrum management has a significant influence on the incidence of diarrhoea and pneumonia and, consequently, calf mortality [19]. The effects of colostrum are widely studied, and significant effects were shown for the use of maternal drenching or mixed colostrum, heating and freezing of colostrum, quantity of colostrum at first feeding, colostrum quality, age of the dam and the vaccination status of the dam [20]. While the quality of colostrum is mainly determined by the health status of the dams and their vaccination prepartum [21], the transfer of immunoglobulins is mainly influenced by time and amount colostrum collection and administration [22]. Analysing calf serum samples for immunoglobulin and total protein concentration allows an assessment of passive immune transfer via colostrum, and determining colostrum density as Brix score is as a good proxy measurement [23–26]. A study in German dairy farms with high calf mortality showed that over 80% of these farms have deficits in colostrum management [16].

During the 1990s, management factors emerged as influential determinants of calf mortality, including herd size, production orientation, drinking practices of preweaning calves, navel disinfection, and the use of antibiotics for calves afflicted with diarrhoea [27]. An important factor with significant impact on calf mortality is the farm staff. There are often differences between farms in terms of the number of people available to care for the calves, the tasks allocated within working hours and the level of expertise [28, 29]. An American study suggested that, apart from staff dedicated to calf care, farm size also holds significance [30]. In comparison to farms with low calf mortality rates, those with higher losses tended to delegate calf care to employees rather than the farmers themselves. However, with increasing farm size, farms where the owner oversaw calf care also exhibited elevated calf losses, suggesting potential neglect of calf health due to more extensive farm operations. Research from Estonia [31], Austria [32], the USA [33], Norway [12] and France [34] corroborates the significant impact of farm size on calf health, thereby influencing mortality rates. French research from 2011 [34] found that increasing herd size correlated with a potential lapse in individual animal care and revealed notable differences in management practices compared to small farms.

Another important factor is the general willingness of the farm to implement measures based on veterinary advice regarding increased calf mortality. According to a study in problem farms in Lower Saxony on acceptance of veterinary advise the farmers justified the lack of willingness to implement measures due to insufficient practicability, the excessive time required and a lack of confidence in their success [16].

In recent years, dairy farming in Europe has undergone considerable change, encompassing both legal requirements and the economic landscape in dairy farming. The number of family farms decreased considerably, and with the expansion of farms the organisational complexities associated with farm and herd management have increased. In parallel, an increasing proportion of milk production is taken by large commercial dairy farms owned by cooperatives or corporates and operated by employees [35]. Therefore, the associations identified by previous studies in family farms over the past decades [12, 27, 34] or by studies from other parts of the world with a different economic and legal framework [33] may not appropriately mirror the current situation in Europe. In order to provide science-based advice the farmers managing growing dairy herds, it is necessary to review the risk factors for calf mortality under current economic, legal and social framework conditions. Thus, the objective of this study was to assess the associations between calf mortality and farm management with special emphasis on calving, newborn care, and colostrum supply as well as calf rearing in large dairy herds.

## Methods

### Farms

As part of a calf health initiative run by the Animal Health Service (TGD) of the Thuringian Animal Disease Fund, 223 Thuringian dairy farms, each housing over 100 dairy cows, were invited to voluntarily take part in calf health monitoring between December 2016 and January 2017. These farms collectively housed 95,812 dairy cows, accounting for 88.9% of Thuringia’s dairy cow population [36]. Of these farms, 98 agreed to participate in this study. Additionally, one farm with fewer than 100 cows was also included in the study, bringing the total number of farms to 99. The study was approved by the Thuringian State Office for Consumer Protection, which is competent authority for research ethics approval in Thuringia, and a formal waiver of the need for animal-use approval was granted (2684-04-5-TSK-19-103).

In the period from March 2017 to March 2018, veterinarians from the Animal Health Service of the Thuringian Animal Disease Fund undertook farm visits and gathered data. During these visits, interviews were conducted with the responsible farm or herd managers using a structured questionnaire. The operational results of this survey were then discussed with those responsible for herd management.

**Fig. 1:**
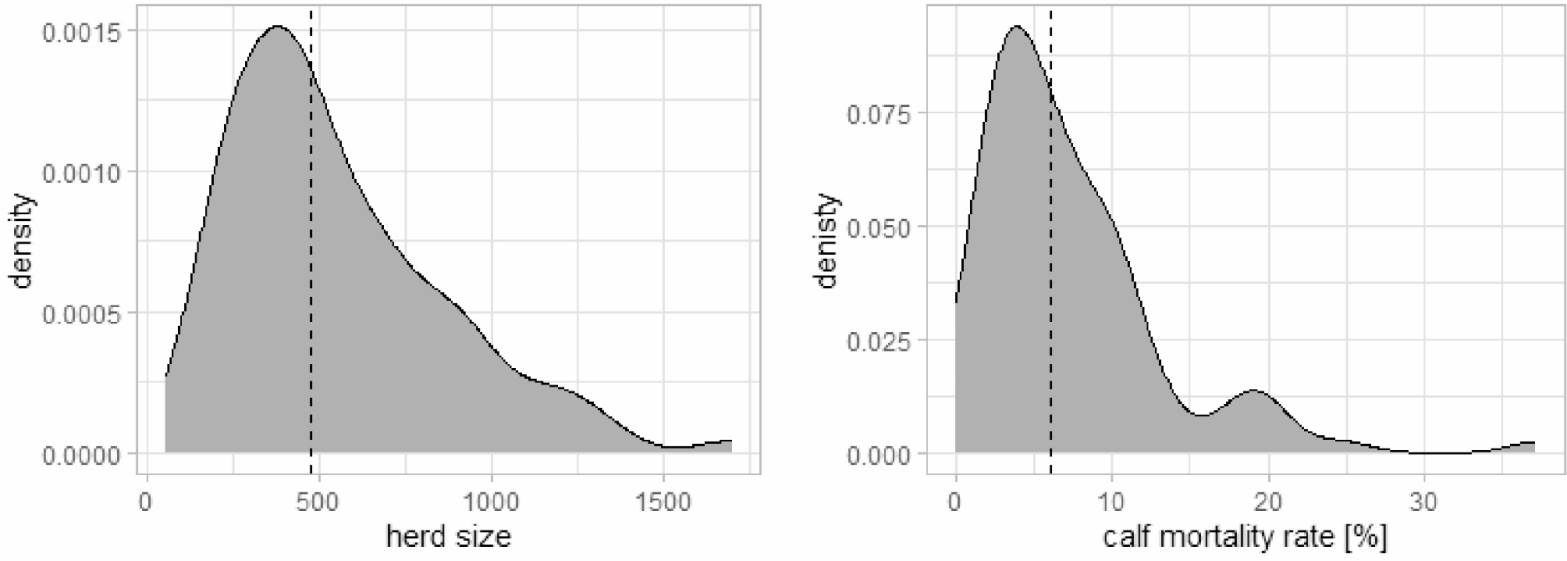
Distribution of (**A**) dairy herd size and (**B**) calf mortality rate for the study herds. Dairy herd size represents number of cattle older than 24 months. Calf mortality rate represents calf losses until the age of 6 months. Dashed line indicates the median value being 474 cows and 5.9% calf mortality rate

Regarding the factors influencing calf mortality, farms that mainly sold their calves to other farms and did not breed their own calves were excluded from the data analysis. Consequently, six farms that predominantly sold their calves to other farms without conducting their own rearing process were omitted from the analysis. This led to a final sample size of 93 farms. The dairy farms included in the analysis were farms which raised their own youngstock. Median size of dairy herds included in the study was 474 cattle over 24 months old (Fig. 1). Across all farms, the cows were kept in loose housing systems. Most cattle represented the German Holstein breed, with smaller proportions of the Simmental, Jersey, and Brown Swiss breeds, or their cross breeds, present in some herds.

### Data collection using a questionnaire

A structured questionnaire was formulated to facilitate data collection, developed by drawing upon the extensive expertise of veterinarians associated with the Animal Health Service. The design of the questionnaire involved crafting specific categories for potential responses, ensuring standardisation in recording various management factors across the farms. Questions included general information on the farm, husbandry, hygiene protocols, and animal care management, as well as information pertaining to colostrum collection and administration, calf health, preventive measures, calving management, and dry cow care. The questionnaire was evaluated by five conveniently selected farms and adjustments were made based on the outcomes. The involvement of seasoned specialist cattle veterinarians, possessing substantial experience in advising dairy farmers, ensured the standardisation of the survey’s breadth through their meticulous development of response categories. This approach allowed for a comprehensive and standardised assessment across diverse farm settings.

Each section of the questionnaire is divided into several individual questions. Interviewees discussed the respective question with the farmer and agreed to choose the answer that best-described calf husbandry management, including birth management practices on the farm. Multiple answers were excluded. The information was verified during a tour of the farm and in rare cases corrected by the interviewer if the farmers assessment of the trait was obviously biased by his own perspective. An overview of the data collected is summarised in Table 1.

**Table 1:** Variables considered for the model with frequencies of categories and *P*-values of the univariable analysis

Variable	Reply category	*n*	ANOVA (*P*-value)
**Herd management**			
Own reproduction	Yes No	90 3	0.015
Sale of male animals during the rearing phase	Yes No	76 16	0.023
**Colostrum supply**			
Time of colostrum administration^1^	Less than 2 h 2 to 4 h more than 4 h NA	35 45 10 3	0.397
Quantity of colostrum administered	Less than 1 L 1 to 2 L More than 2 L NA	0 17 73 3	0.938
Time of colostrum collection	Less than 2 h 2 to 4 h more than 4 h NA	28 47 17 1	0.685
Provision of dam-sourced colostrum	Yes No	64 29	0.036
**Calf feeding**			
Whole milk drinking trough in group housing	Yes No	11 82	0.088
Milk replacer with plant-based components	Yes No	43 34	0.094
Total mixed ration as solid feed	Yes No Other solid feed	40 44 7	0.072
Disinfection of trough teat	After each use Daily Less often Never	0 22 45 26	0.050
Trough teat replacement	Severe wear Minor indications of wear^2^	50 43	0.004
Automatic feeder teat replacement	Severe wear Minor indications of wear Certain time interval No milk dispenser	52 30 7 4	0.073
**Calf housing**			
Cleaning of group housing	After each dung removal Before every new occupancy Less often Never	22 44 25 0	0.007
Dung removal in individual housing	Up to every 5 days^3^ Less often	9 81	0.267
Dung removal in group housing	Daily Every two days Every 2–5 days Less often	9 9 10 63	0.238
Disinfection of group housing	After each cleaning Before every new occupancy Less often Never	15 39 27 10	0.010
**Health-related factors**			
Regular occurrence of diarrhoea in the first two weeks of life	Yes No	68 25	0.052
Regular occurrence of respiratory diseases in wintertime	Yes No	53 40	0.061
Regular occurrence of respiratory diseases all year	Yes No	61 32	0.005
Dust as cause of respiratory diseases	Yes No	23 70	0.011
Regular occurrence of navel disease	Yes No	60 33	0.010
Regular occurrence of trichophytia	Yes No	26 67	0.051
Regular occurrence of calf diarrhoea	Yes No	61 32	0.188
Estimated losses due to respiratory disease	0–25		< 0.001
Estimated losses due to navel disease	0–8		0.651
Estimated losses due to diarrhoea	0–25		0.001
**Calving and preparation of cows**			
Maternity vaccination with annual booster	Yes Without boostering No vaccination	47 10 36	0.083
Assessment of body condition via	Subjective evaluation^4^ objective evaluation^5^	75 18	0.046
Separate calving box for sick cows	No sick cows Always identical box Temporary or not	12 19 62	0.030
Hygiene of the walls of the calving box	Clean Slight incrustation on walls Smeared with faeces	27 57 9	0.091
Hygiene of feeding container for suckling calves	Clean Slight incrustation on walls Smeared with faeces	69 23 1	0.011

^1^ The variable “time of colostrum collection” was correlated to the variable “time of colostrum administration”, and therefore not included into the multivariable model

^2^ includes the categories “minor indications of wear”, (*n* = 37) and “regular time intervals” (*n* = 6)

^3^ includes the categories “daily” (*n* = 1), “very two days” (*n* = 1) and “3–5 days” (*n* = 7)

^4^ includes the categories “subjective evaluation” (*n* = 74), and “none” (*n* = 1)

^5^ includes the categories “Body condition score” (*n* = 14), and “Back fat thickness” (*n* = 4)

The calf mortality rate was calculated by assessing the deaths of calves within the first six months of their lives relative to the total number of calves alive at birth. This calculation relied on the data derived from farm reports submitted to the German Database for Animal Movement (HI-Tier) for the year 2017. In Germany, cattle farmers are mandated to report all herd additions, including births, within seven days of the cattle’s arrival, specifying the ear tag numbers for both the calf and its mother. Similarly, departures from the herd, whether due to sale, slaughter, or death, must also be recorded with reasons provided. The pertinent information extracted from the database included the total number of births in 2017, serving as the denominator in the calculation. Additionally, the numerator was determined by the count of calves reported as deceased before reaching the age of six months in 2017. It is important to note that stillborn calves are not included in these records, as their status does not necessitate reporting, as neither a birth nor an addition to the herd is required for these cases.

### Statistical analysis

The data collected from the questionnaires and the calf mortality rates extracted from the German Database for Animal Movement were transcribed into a Microsoft Excel spreadsheet (Microsoft Corporation, Redmond, Washington, USA). In instances where certain categories within variables were underrepresented or lacked any data, these findings were condensed for better comprehension. Specifically, categories with frequencies of four or fewer were combined into two meaningful groups to ensure frequencies of five or more for further analytical purposes. For the subsequent analysis, statistical software R 3.6.2 [37] was used for both descriptive analysis and the formulation of statistical models.

The possible influence of the surveyed management factors and herd size as independent variables on calf mortality (dependent variable) was calculated using a univariable risk factor analysis (analysis of variance; ANOVA). To create a generalised linear mixed model using the lme4 package (version 1.1–21) for a causal analysis, all risk factors suggesting a possible association with calf losses in the ANOVA (i.e. factors yielding *P* < 0.1) and significant factors from previously published studies were selected. The Akaike Information Criterion (AIC) was then used to assess whether an exclusion of factors resulted in an improvement of the model. The exclusion and reintroduction of variables was carried out until further variation (exclusion or reintroduction of variables) did not result in further improvement of the model. The significance level for the independent variables was set at *P* < 0.05. To assess collinearity among factors within the multivariable model, the variance inflation factor was computed using the “car” package (version 3.0–6).

## Results

Data from 93 Thuringian dairy farms were included in the analysis. Of the 93 farms, 74 kept pure dairy herds (79.57%), and 19 farms (20.43%) focussed on at least one other production specialisation in addition to dairy farming. Herd size was not homogenously distributed across the farms.

**Fig. 2:**
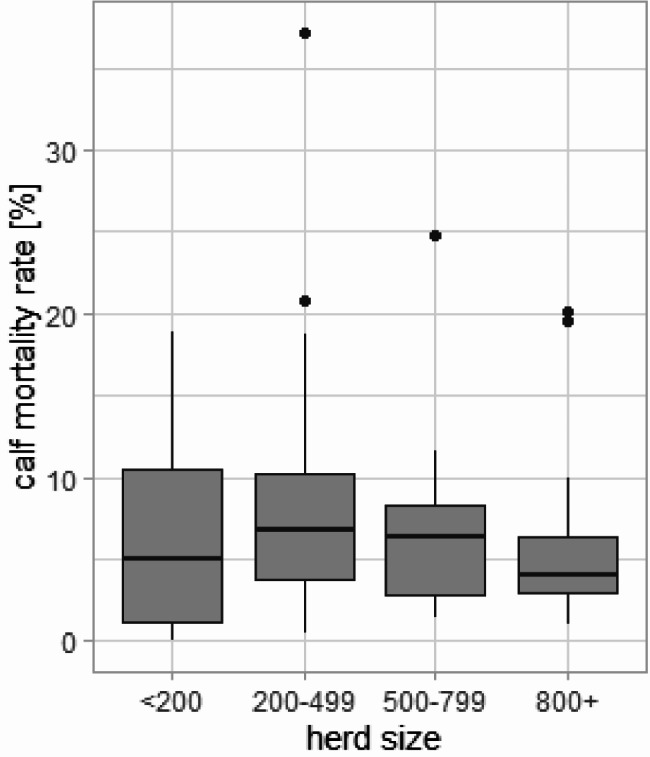
Boxplot showing the association between herd size and calf mortality

According to the data from the HI-Tier database of the 93 study herds from 2017, 54,474 calves were born alive and 3,790 calves died within the first six months of life. Thus, calf losses within the farms analysed varied between 0.0% and 37.2%, with a mean calf mortality rate of 7.3% and a median of 5.9% (Fig. 1). Using ANOVA, no statistically significant correlation was detected between herd size and calf mortality among these farms (Fig. 2). Herds with fewer than 200 cows had a median calf mortality rate of 5%, herds with 200–499 cows a median of 6.75%, herds with 500–799 cows a median of 6.4% and herds with 800 or more cows a median of 4%.

The univariable ANOVA highlighted 21 factors with a *P*-value below 0.1, indicating their potential association with calf mortality (Table 1). These 21 factors and a further 11 that had a significant effect on calf mortality in previous studies were initially incorporated into a multivariable model. Because the univariable analysis did not identify any statistically significant association between herd size and calf mortality, the variable herd size was not included into the multivariable model. The multivariable model was subsequently refined to 15 factors using the AIC for model enhancement. These 15 factors were eventually integrated into the final generalised linear mixed model (as presented in Table 2). From this comprehensive multivariable analysis, four variables stood out as significantly correlated with calf mortality, encompassing aspects related to calf health, drinking management, and hygiene practices (as illustrated in Table 3). Among the health-related variables, the presence of dust contributing to respiratory disease (regression coefficient β=-0.25, *P* = 0.005) exhibited significant relationships with calf mortality. In terms of drinking management, the act of calves consuming their mother’s colostrum (β = 0.22, *P* = 0.007) emerged as a significant factor. Additionally, concerning hygiene practices, regularly replacing the trough teat (β=-0.21, *P* = 0.003) was identified as a significant influencing factor impacting calf mortality. Furthermore, in herds where the management practice of scoring the dam’s prepartum body condition with objective methods (body condition scoring or measurement back fat thickness), calf mortality was significantly lower than in herds where this practice was not implanted or done only subjectively (β=-0.2, *P* = 0.025). When determining the body condition, 79.6% (*n* = 74) of the farmers stated that this was determined by subjective assessment, on 14 farms (15.1%) body condition scoring was implemented, and on four farms (4.3%) back fat thickness was measured by sonography. In one farm the body condition of prepartum cows was not estimated at all.

**Table 2:** Linear mixed model analysing management factors in correlation with calf losses

Predictors	Estimates	Confidence Interval	*P*-value	Wald-Test
(Intercept)	0.89	0.46–1.32	< 0.001	
Time of colostrum collection				0.1883
• Less than 2 h • 2 to 4 h • Over 4 h	Reference -0.10 -0.20	-0.27–0.07 -0.43–0.02	0.227 0.071	
Quantity of colostrum given				
• 1 to 2 L • More than 2 L	Reference -0.03	-0.19–0.13	0.690	
regularly occurring respiratory diseases				
• Yes • No	Reference -0.10	-0.25–0.05	0.180	
regularly occurring diarrhoea				
• Yes • No	Reference -0.07	-0.22–0.08	0.383	
Dust as cause of respiratory diseases				
• yes • No	Reference -0.25	-0.41 – -0.08	**0.005**	
Trough teat replacement				
• severe wear • minor indications of wear	Reference -0.21	-0.35 – -0.07	**0.003**	
Provision of dam-sourced colostrum				
• Yes • No	Reference 0.22	0.06–0.39	**0.007**	
Mortality due to diarrhoea	0.03	-0.00–0.05	0.054	
Mortality due to lung disease	0.01	-0.02–0.04	0.496	
Mortality due to navel disease	0.06	-0.00–0.12	0.051	
Whole-milk troughs for suckling calves in group housing				
• Yes • No	Reference 0.03	-0.20–0.25	0.816	
Dung removal in individual housing				
• Less often • Up to every 5 days	Reference 0.20	-0.02–0.43	0.076	
Dung removal in group housing				0.3634
• daily • every two days • every 2–5 days • less often	Reference -0.02 -0.15 0.07	-0.35–0.31 -0.49–0.19 -0.22–0.35	0.904 0.375 0.651	
Disinfection of group housing				0.1834
• after each cleaning • before every new occupancy • less often • never	Reference 0.03 0.19 0.08	-0.16–0.21 -0.01–0.39 -0.21–0.37	0.783 0.068 0.584	
Assessment of body condition				
• subjectively • objectively	Reference -0.20	-0.37 – -0.03	**0.025**	

**Table 3:** Significant management factors, median, mean, 95% confidence interval for calf mortality in thuringian dairy farms

Factor	Median	Mean value	95% Confidence Interval	*P*-value
Dust as cause of respiratory diseases				
• Yes • No	8.7 4.7	9.9 6.5	6.0-11.2 2.9-8.9	0.005
Trough teat replacement				
• severe wear • minor indications of wear	6.8 4.2	9.0 5.4	4.1-10.1 2.4-7.3	0.003
Provision of dam-sourced colostrum				
• Yes • No	5.8 6.3	7.3 7.3	3.0-9.9 4.1-10.1	0.007
Assessment of body condition				
• subjectively • objectively	6.4 3.9	7.9 4.9	3.6-10.1 2.2-6.3	0.025

Among the farm managers surveyed, 23.9% (*n* = 23) considered increased dust exposure in the herd to be a cause of respiratory disease in calves. On these farms, the mean calf mortality rate stood at 10.2% (with a median of 8.8%). Conversely, among farms that did attribute respiratory disease primarily to dust but rather suspected other factors such as draughts, harmful gases or infectious agents as the primary cause of pneumonia in their calves (*n* = 70), the calf mortality rate averaged 6.5% (median 4.65%).

Among the 93 dairy farms surveyed, 56 farms (60.2%) reported providing their new-born calves with dam-sourced colostrum for their first feed. These farms displayed an average calf mortality rate of 6.2% (median 4.9%). The remaining 39.8% of the farms (*n* = 37) did not take maternal origin into account when allocating colostrum. Within this group, the average calf mortality rate was higher at 8.9% (median 6.7%).

In examining drinking hygiene practices, the replacement of teats was scrutinised, offering respondents the choice between ‘significant wear’ and ‘slight wear’ for both bucket troughs and the automatic troughs. Over half (53.8%; *n* = 50) of the farm managers stated that they only replaced the teats of the bucket when they were clearly worn. The average calf mortality rate on these farms stood at 9.0% (median 6.8%). A teat replacement at slight indication of wear was practised by 46.2% of the farms (*n* = 43), with calf mortality averaging at 5.4% (median 4.2%).

When the farm managers were asked to estimate the proportion of calf losses attributed to diarrhoea or navel disease, responses ranged from 0 to 25% for diarrhoea and 0–8% for navel disease. In our model, these two variables slightly missed significance level for a positive association with calf mortality rate showing only minor impact (diarrhoea: (β = 0.03, *P* = 0.054); navel disease: (β = 0.06, *P* = 0.051)).

## Discussion

The primary objective of this study was to analyse dairy farms, focussing on their farm management practices, specifically in the domains of colostrum supply, calf husbandry, calf care, and hygiene management. The aim was to discern potential correlations between these practices and calf mortality rates. Additionally, the study sought to explore the potential relationship between calf mortality rates and herd size. The analyses revealed clear correlations between supply of dam-sourced colostrum, hygiene of bucket teats, dust contributing to respiratory diseases, and calf losses.

### Calf mortality and herd size

The univariable analysis did not discern any statistically significant relationship between herd size and calf mortality. The wide-ranging calf mortality rates observed across the sample (0.0−37.2%) strongly suggest that factors other than herd size independently influence calf losses. Comparable variations among farms were previously noted in Thuringian dairy farms during 2006/2007 with a variation form 0.0-25.9% [17]. A study in dairy farms in North-East Germany [13] and a more recent investigation in the same region published in March 2022 [38] similarly failed to establish a direct correlation between herd size and calf mortality. These studies also concluded that herd size did not significantly influence calf mortality rates.

One notable strength of our study lies in the large number of participating farms with herd sizes up to 1,700 cows (median 474 cows) that are common for commercial dairy farms operated with employees. It can be assumed that typical management practices which are characteristic of such dairy herds were recorded. Importantly, the sample was not solely restricted to problematic farms; only those farms not engaged in the rearing of their own offspring were excluded from the study. This approach ensured an ample number of farms for conducting a thorough analysis of relationships pertaining to calf health within a multivariate model. However, it should be noted that a limitation in our study related to the non-random selection of farms. It is plausible that farms aware of inadequacies in their calf management practices may have chosen not to participate in the study, and therefore may be underrepresented.

A further limitation is the unavailability of individual-based calf management data; instead, information was gathered based on standard practices within the herd. Conducting a survey based on individual animals necessitates considerably more effort and would have been feasible only with a much smaller sample size. Nonetheless, this study aimed to include as many farms as possible to attain results that are generalisable and pertinent to the broader study population.

Larger farms typically adhere to established protocols, particularly concerning the provision of dam-sourced colostrum, as well as trough teat replacement. Additionally, these results can be extrapolated to dairy cattle populations with similar structures, specifically larger herds on farms primarily managing their own breeding.

### Management factors relating to calf health

Dust serves as a visible indicator of an inadequate barn climate. Functioning as a mechanical irritant, it adversely affects animal welfare, human health, and farm technology. As a carrier and nutrient medium for microorganisms as well as fungal and bacterial toxins, dust can penetrate the alveoli of the lungs, depending on the particle size, and be a precursor for respiratory diseases [39, 40]. This is also confirmed by a recent Belgian study [41], in which dust was identified as a carrier of particulates and endotoxins and was linked to pneumonia in calves. Increased exposure of the airways to fine dust particles can overwhelm the organism’s ability to eliminate them via the mucociliary clearance system and thus trigger an increased influx of phagocytic alveolar macrophages, cytokine secretion and neutrophil migration, causing airway inflammation [41]. In addition to acute and chronic respiratory disease can also be the result of excessive dust inhalation, as has been described in horses with dust-induced asthma [42]. Together with other management factors such as harmful gases, increased temperature, unsuitable humidity levels, or draughts, dust was identified as a predisposing factor to respiratory disease. These issues arise from poorly ventilated stables and can act as additional stressors, compromising the immune system’s functionality [41, 43]. Enhancing barn ventilation is crucial for fostering a healthy barn environment. An American study suggested reducing harmful dust particles by filtering the barn air [44]. A previous German study identified an association between the frequency of antibodies against bovine respiratory syncytial virus or parainfluenca-3 virus and farm managers suspected dust as a contributing factor to calf respiratory disease [17].

Nonetheless, risk factors for respiratory diseases are complex, and the statement of the farmer regarding the role of dust may have been caused by his education and knowledge about dust as a risk factor. Nevertheless, for optimal animal welfare and species-appropriate housing, an alternative approach to minimise dust exposure might involve providing proper outdoor housing for calves in igloos or ensuring ample fresh air supply coupled with low-dust bedding.

According to our analysis, bedding management was not found to be significantly associated with calf mortality rate. However, the variable ‘Dung removal in individual housing’ sightly missed significance level suggesting that there may be a relationship between the frequency of dung removal and the calf mortality rate. Farms with a manure removal interval of up to every 5 days tended to have higher calf loss rate than farms that stated that they removed manure less frequently than 5 days. This may be due to the fact, that the farms with a more frequent manure removal frequency are aware of the importance of this measure and, in the event of increased calf losses, are already trying to counteract the problem. Because the variables ‘Dung removal in individual housing’, ‘Dung removal in group housing and ‘disinfection of group housing’ were included in the final model, we controlled for interactions with these management practices and improved the validity of our model.

In a study from Italy [45], long renewal intervals in bedding management, which lead to increased soaking of the bedding, were associated with an increase in intestinal pathogenic microorganisms in the pen air and an increased incidence of calf cough. Maintaining a low litter humidity can help to improve the microbiological air quality in calf pens and thus reduce morbidity and mortality rates in calves. A French study identified an increased the risk of regarding umbilical infections with humidity of the bedding as a significant risk factor [46].

Farm managers’ estimates of the prevalence of diarrhoeal diseases were positively correlated with actual calf mortality rates, underscoring the widely acknowledged association between diarrhoeal diseases and calf deaths. Historically, losses attributed to diarrhoeal diseases were believed to be approximately twice as high as those attributed to respiratory diseases [13]. However, a cross-sectional study conducted in north-east German dairy farms in 2016 revealed a lower incidence at herd level for neonatal calf diarrhoea (median 12.0%) compared to respiratory disease (median 17.5%) [13]. The authors did not establish a direct relationship with calf losses; however, they observed a correlation with the administration of halofuginone, a therapeutic agent used in cases of frequent cryptosporidiosis in the herd. Cryptosporidium parvum infections represent the most prevalent cause of calf diarrhoea [13].

As a complex factor disease, calf diarrhoea may arise from infectious causes while also being influenced by feeding practices, housing conditions, and hygiene management [1, 13]. Moreover, prompt identification and intervention are crucial for positive treatment outcomes. This requires dedicated and well-trained staff, as well as adequate animal observation time [28, 29]. Implementing preventive measures, such as maternity vaccinations, can significantly reduce the occurrence of neonatal diarrhoea in calves.

According to previous studies, body condition of dams is a significant factor that can influence the calf mortality rate. According to a study from Germany, lean animals with a body condition score below 3.0 were found to have a higher risk of dystocia [47]. In a French study, a body condition score above 4.0 was associated with an increased risk of stillbirths [48]. Dystocia is known not only to pose a significant risk to the dams, but also to affect the risk of stillbirths and births of weak calves [49, 50]. Hypoxia and acidosis caused by dystocia or trauma to the calf caused by improper obstetrics significantly impacts calf mortality rate [49, 51]. Therefore, the prepartum body condition of the dam can indirectly influence the calf mortality rate and should be monitored as part of on-farm health management. For this purpose, objective measurements represent a more valid result than those based on subjective assessment by the farmer, as they are determined using a standardized procedure and are comparable across farms. Our study revealed that the objective determination of body condition by BCS scoring (14 herds) or sonographic measurement of back fat thickness (four herds) was significantly associated with lower calf mortality rate compared to subjective assessment by the farmer, probably reflecting a high general level of herd management.

### Feeding management – dam-sourced colostrum

The vital importance of supplying calves with immunoglobulins via colostrum has been widely acknowledged and remains a subject of extensive research [25, 52]. Key components include immunoglobulins, leukocytes, growth factors, hormones, non-specific antimicrobial factors, and nutrients. Ensuring proper drinking management is vital for the effective transfer of these essential elements for calves to adequately absorb them [22]. In a study conducted in northeast Germany, a notable prevalence of calves with inadequate immunoglobulin transfer was associated with increased on-farm calf mortality of over 5%. In problem farms in Lower Saxony, where calf losses exceeded 20%, a substantial 80% of the cases revealed inadequate passive transfer of immunity through colostrum [16]. Beyond the essential components and feeding regime, the quality of colostrum holds significance. This quality is not only impacted by the dam [21] but also by various management factors. An Austrian study conducted in 2015 [32] described calf diarrhoea as the most prevalent clinical issue on the farms investigated. This issue was associated with subpar milk and colostrum quality, as 84.1% of the farms reported using milk obtained from sick cows (such as those with mastitis, elevated somatic cell counts, and cows undergoing medication treatment). Additionally, these farms also utilised frozen colostrum for feeding calves [32]. Monitoring the quality of colostrum is a high priority in well-managed dairy farms and several methods are widely used ranging from measuring density or viscosity of colostrum to laboratory-based methods [26]. In particular, the determination of the serum IgG content in calves within the first days of life enables a concrete statement to be made about the success of the passive immune transfer and thus also provide a quality assessment of the farm’s colostrum management. According to current recommendations, the minimum serum IgG content for a successful immune transfer is 10 g/L [11, 23]. Calves with serum levels below this threshold are at risk for higher mortality rate. However, a serum IgG level of at least 25 g/L is considered the standard target for optimal care in order to significantly reduce both the mortality rate and the morbidity rate of calves [23]. As such measurements are associated with significant additional work for a farm, at least the Brix refractometry of the colostrum should be performed regularly. Brix values are well correlated with serum immunoglobulin concentration [53, 54].

In our study, we considered primary aspects concerning colostrum management, including the timing of colostrum collection and intake and quantity of intake. Surprisingly, except for maternal colostrum feeding, all other factors exhibited no significant impact on the calf mortality rate. As shown in a previous study based on data of the Thuringian calf health monitoring with special emphasis on immunoglobulin transfer [22] and as widely accepted [23], these factors are associated with the serum concentration of immunoglobulins and total protein in the calves’ serum. This suggests that apart from the supply of immunoglobulins to calves, additional factors might also contribute to calf losses, and one of those is the use of dam-sourced colostrum at first feeding.

The farms with a dam-sourced feeding approach exhibited a notably lower calf mortality rate, potentially linked to the presence of maternal leukocytes in the colostrum. Various studies have highlighted the significance of maternal leukocytes, particularly macrophages and lymphocytes, for calf health [25]. These maternal leukocytes exhibit immunological activity not only in the calf’s intestine upon oral intake but also enter the neonatal bloodstream and accumulate in diverse organs, thereby regulating the calf’s immune system [25].

It has been demonstrated that only the dam’s leukocytes are absorbed by the neonatal organism. Calves supplied with maternal colostrum seem capable of generating antibodies more swiftly and expediting the development of their immune system. Consequently, they may possess better protection against infections and display reduced or less severe disease progression than calves that have not been provided maternal colostrum [19]. A study in pigs reported similar findings, where piglets ingesting their mother’s colostrum exhibited enhanced cellular immunity [55].

Moreover, if calves are fed dam-sourced colostrum, the colostrum is likely to be harvested soon after the cow has calved. This may have improved colostrum supply in two major aspects with the effect that the calf was fed sooner and thus be able to absorb more [20, 56] and the cow will be milked sooner after calving ensuring a high concentration of immunoglobulins in the colostrum [57, 58].

Additionally, substantial distinctions have been observed between calves consuming native colostrum and those fed heated or frozen, cell-free colostrum. Significant differences have been observed between calves that ingest native colostrum and calves that have been fed temperature-treated (heated or frozen), cell-free colostrum. The disparity is evident in the rate and composition of monocytes and the response of B and T cells post-vaccination: calves fed cell-free colostrum displayed lower monocyte levels in the blood and generated fewer B and T cells after vaccination [59–61]. Consequently, calves receiving maternal colostrum develop their immune system more rapidly compared to those provided with leukocyte-free colostrum [20]. It is presumed that leukocytes actively traverse the intestinal wall, a process only viable with intact, living cells. Temperature-treated lose their ability to actively pass through the intestinal barrier and thus remain unabsorbed, emphasising a need for consideration when storing colostrum in the future.

The authors assume that feeding non-maternal colostrum might be a typical management practice particularly on larger dairy farms. In small farms with fewer simultaneous births, usually, only maternal colostrum is available to the calf. However, as herd size increases and consequently leads to a higher birth rate, cattle farmers often resort to providing frozen colostrum from other mothers or mixed colostrum from cows calving on the same day, citing various reasons [32]. The process of separately milking fresh calvers and feeding the calves with colostrum from their respective mothers demands more labour and may not always be feasible or advisable, particularly if the dam is unwell. Additionally, in the context of paratuberculosis control, it is recommended to administer initial colostrum from cows that have tested negative for calves born to mothers that have tested positive [62]. Concerns about heifer colostrum, often perceived as inferior in quality (particularly in terms of immunoglobulin content) compared to cow colostrum, also influence the decision to opt for mixed colostrum. However, based on our findings and considering the insights into barn-specific maternal immune systems, we argue that incurring this additional expense is justified, particularly when implementing maternal vaccinations.

### Hygiene management - replacing the trough teats

In the herds examined in this study, calves were predominantly fed using a teat trough (bucket or automatic feeder) from the initial feeding until weaning age. The teat of a trough serves as a substitute for the maternal teat and marks one of the earliest points of contact between a calf and its surroundings. Considering that infectious agents, especially those causing diarrhoeal diseases, are primally ingested orally [13], it can be inferred that the hygiene of a teat might significantly impact calf health. As shown previously, hygiene of feeding equipment reduces diarrhoea and septicaemia in newborn calves [63]. With respect to artificial trough teats, a recent German study showed that the highest bacterial load found was not in the housing area, but in feeding equipment, especially inside the teats of milk feeding buckets [64]. This finding underlines the importance of the hygienic condition of teats and is consistent with our results that farms that only replaced severely worn teats exhibited notably higher calf loss rates compared to those already replacing the teats when slightly worn. Material wear on teats is influenced by the frequency and intensity of use, resulting in surface roughening, abrasions, and cracks. These surface alterations, combined with organic substances like dirt, saliva, and milk residues, create an ideal environment for microflora proliferation and biofilm formation. Although cleaning and disinfecting the teat can reduce bacterial presence, this effectiveness is limited by the extent of the wear and tear, particularly the depth of grooves and cracks. Furthermore, more worn-out teats may leak milk which could hamper calves’ ability to drink.

Bacterial load of colostrum is significantly associated with lower serum immunoglobulin concentrations of calves [65]. In a Canadian study, in most farms total bacterial count measured in colostrum feeders exceeded the threshold for drinking milk of 100,000 colony forming units/mL [66–68]. Implementation of hygiene measures on feeders varies greatly from farm to farm with respect to cleaning frequency, water temperature and the use of cleaning agents [32]. The poorly accessible areas inside the artificial teat, which has the highest bacterial load [64], are a limiting factor for a hygienic cleaning measure that requires removing of the teat from the bucket [69]. Unless visually perceptible cleanliness does not automatically correspond to the desired hygienic condition and visual hygiene status alone is not a suitable indicator for assessing bacterial contamination, visual assessment of cleanliness is important to optimise the desired effect of cleaning agents and disinfectants, which would already be reduced by visually perceptible dirt deposits [70]. In addition, bioluminescence swab methods would allow a fast and on-site assessment of the degree of pollution [67].

Additionally, the contamination of the teat with the individual calf’s microflora during the drinking process should not be overlooked. Furthermore, the direct transmission of infectious agents, especially diarrhoea pathogens, is possible if the buckets or automatic troughs are not clean. We hypothesise that regularly cleaned teats, replaced after moderate wear, could positively impact calf health by reducing the presence of pathogens. Therefore, clearly defined working processes, easily removeable teats, and implementing a consistent interval for each replacement, may aid in lowering calf mortality rates. This highlights the importance of establishing routines to standardise farm hygiene management, particularly on larger farms. The results can be extrapolated to dairy cattle populations with similar structures, specifically larger herds on farms primarily managing their own breeding.

## Conclusions

Our analysis of 93 dairy farms in Thuringia has validated the substantial impact of farm management on calf mortality. Regardless of farm size, our findings indicate correlations with management practices concerning colostrum administration, hygiene of bucket teats, and dust. Feeding dam-sourced colostrum can significantly boost calf immunity and is as equally crucial as ensuring adequate immunoglobulin transfer, thereby considerably reducing calf mortality rates. These practices demand consistent implementation, along with appropriate additional resources in terms of time, workforce, and technology for calf husbandry. Implementing measures to mitigate excessive dust exposure could notably decrease calf losses linked to respiratory disease. Modern dairy cow facilities, designed to meet both elevated structural requirements and the animal welfare demands set by contemporary society and regulations, offer potential for improvement in this regard. These facilities often provide better opportunities to streamline operational processes using modern technical equipment. The time saved through such advancements should be dedicated to thorough animal observation and individualised care by well-trained and dedicated staff.

## Data Availability

The datasets analyzed during the current study are available from the corresponding author on reasonable request.

## Declarations

### Prior publication

The data present herein have not been previously published.
